# Genetic Evaluation of A Nation-Wide Dutch Pediatric DCM Cohort: The Use of Genetic Testing in Risk Stratification

**DOI:** 10.1161/CIRCGEN.120.002981

**Published:** 2022-09-30

**Authors:** Marijke H. van der Meulen, Johanna C. Herkert, Susanna L. den Boer, Gideon J. du Marchie Sarvaas, Nico A. Blom, Arend D.J. ten Harkel, Hans M.P.J. Breur, Lukas A.J. Rammeloo, Ronald B. Tanke, Carlo Marcelis, Ingrid M.B.H. van de Laar, Judith M.A. Verhagen, Ronald H. Lekanne dit Deprez, Daniela Q.C.M. Barge-Schaapveld, Annette F. Baas, Arjan Sammani, Imke Christiaans, J. Peter van Tintelen, Michiel Dalinghaus

**Affiliations:** Dept of Pediatric Cardiology, Erasmus MC, Univ Medical Center Rotterdam, Rotterdam (M.H.M., M.D.); Dept of Genetics, Univ of Groningen, Univ Medical Center Groningen, Groningen (J.C.H., I.C.); Dept of Pediatric Cardiology, Univ of Groningen, Univ Medical Center Groningen, Groningen (G.J.M.S.); Dept of Pediatric Cardiology, Univ of Leiden, Leiden Univ Medical Center, Leiden (N.A.B., A.D.J.H.); Dept of Pediatric Cardiology, Univ of Amsterdam, Academic Medical Center, Amsterdam (N.A.B.); Dept of Pediatric Cardiology, Univ of Utrecht, Wilhelmina Children’s Hospital, Univ Medical Center Utrecht, Utrecht (H.M.P.J.B.); Dept of Pediatric Cardiology, Amsterdam Univ Medical Center, location Free Univ Medical Center, Amsterdam (L.A.J.R.); Dept of Pediatric Cardiology, Radboud Univ Medical Center, Nijmegen (R.B.T.); Dept of Genetics, Radboud Univ Medical Center, Nijmegen (C.M.); Dept of Clinical Genetics, Erasmus MC, Univ Medical Center Rotterdam, Rotterdam (I.M.B.H.L., J.M.A.V.); Dept of Clinical Genetics, Amsterdam Univ Medical Center, location AMC, Amsterdam (R.H.L.D., J.P.T.); Dept of Clinical Genetics, Univ of Leiden, Leiden Univ Medical Center, Leiden (D.Q.C.M.B-S., I.C.); Dept of Genetics, Univ of Utrecht, University Medical Center Utrecht, Utrecht, the Netherlands (A.F.B., A.S., J.P.T.)

**Keywords:** cardiomyopathy, dilated, genetic testing, pediatric cardiology

## Abstract

This study aimed to describe the current practice and results of genetic evaluation in Dutch children with dilated cardiomyopathy and to evaluate genotype-phenotype correlations that may guide prognosis.

**Methods:**

We performed a multicenter observational study in children diagnosed with dilated cardiomyopathy, from 2010 to 2017.

**Results:**

One hundred forty-four children were included. Initial diagnostic categories were idiopathic dilated cardiomyopathy in 67 children (47%), myocarditis in 23 (16%), neuromuscular in 7 (5%), familial in 18 (13%), inborn error of metabolism in 4 (3%), malformation syndrome in 2 (1%), and “other” in 23 (16%). Median follow-up time was 2.1 years [IQR 1.0–4.3]. Hundred-seven patients (74%) underwent genetic testing. We found a likely pathogenic or pathogenic variant in 38 children (36%), most often in *MYH7* (n = 8). In 1 patient initially diagnosed with myocarditis, a pathogenic *LMNA* variant was found. During the study, 39 patients (27%) reached study endpoint (SE: all-cause death or heart transplantation). Patients with a likely pathogenic or pathogenic variant were more likely to reach SE compared with those without (hazard ratio 2.8; 95% CI 1.3–5.8, *P* = 0.007), while transplant-free survival was significantly lower (*P* = 0.006). Clinical characteristics at diagnosis did not differ between the 2 groups.

**Conclusions:**

Genetic testing is a valuable tool for predicting prognosis in children with dilated cardiomyopathy, with carriers of a likely pathogenic or pathogenic variant having a worse prognosis overall. Genetic testing should be incorporated in clinical work-up of all children with dilated cardiomyopathy regardless of presumed disease pathogenesis.

Since the early 1990s, gene variants have been implicated in the pathogenesis of dilated cardiomyopathy (DCM), which is defined as systolic dysfunction and increased ventricular chamber volume. Genetic DCM was initially thought to be primarily caused by variants in genes encoding cytoskeletal and sarcomeric proteins.^1–3^ However, recent advances in sequencing and array-based technologies have increased our understanding of the genetic basis of DCM. In addition to genes encoding sarcomeric and cytoskeletal proteins, genes coding for transcription factors, ion channels, the nuclear membrane, and mitochondrial proteins are now also known to be involved in isolated DCM. In addition, >200 genes are known that underlie syndromes or inborn errors of metabolism in which DCM can be part of the phenotype.^4–6^

As in adult-onset cardiomyopathy, genetic testing has now been integrated into daily clinical practice in the pediatric population, and a genetic cause can be identified in up to 27%–54% of pediatric DCM patients.^7–9^ *myosin heavy chain 7 (MYH7*) (5.1%), *vinculin (VCL*) (3.2%), and *tropomyosin1 (TPM1*) (2.2%) are among the most frequently affected genes in children younger than 2 years of age, whereas *titin (TTN*) (10.0%), *RNA-binding motif 20 (RBM20*) (6.7%), and *troponin T2 (TNNT2*) (4.7%) are the most frequently mutated genes in the 2–18 year age group.^10^

The pathogenesis of pediatric DCM is a strong predictor of long-term outcome. The 5-year transplant-free survival rate is 47% in idiopathic DCM, whereas it is 73% in DCM related to myocarditis. In familial DCM, the 5-year survival rate is high (94%), but the 5-year transplantation rate is also relatively high (38%). These differences emphasize the importance of establishing the genetic cause in DCM, as it may help further guide optimal treatment.^11,12^ Studies in adult DCM patients have reported a more severe phenotype and earlier onset in patients with a pathogenic genetic variant compared with variant-negative patients.^13,14^ Furthermore, DCM patients with pathogenic variants in *lamin A/C (LMNA), phospholamban (PLN*), *RBM20, desmin (DES)*, and *filamin-C (FLNC*) are at higher risk for malignant arrhythmias and have a worse prognosis than patients with variants in other genes.^13–16^ Matthew et al showed that the affected gene (eg, *MYH7*), a higher variant burden, and *de novo* variant status are all factors independently associated with earlier onset and higher frequency of adverse outcomes in pediatric hypertrophic cardiomyopathy.^17^ However, studies reporting on the utility of genetic testing for risk stratification in children with DCM are scarce.

The aims of the present study were 2-fold. First, we aimed to describe the current practice and results of genetic evaluation in a large cohort of pediatric DCM patients presenting to all tertiary referral hospitals in the Netherlands. Second, we evaluated these patients for potential genotype–phenotype correlations that may guide prognosis.

## Methods

The data, analytic methods, and study materials will not be made available to other researchers for purposes of reproducing the results or replicating the procedure to protect patient privacy. The data that support the findings of this study are available from the corresponding author upon reasonable request. This study was approved by the Medical Research Ethical Committee of the Erasmus Medical Center (MEC 2014-062). All legal parents and children ≥12 years of age gave their written informed consent. Detailed methods are available in the Supplemental Methods.

## Results

### Patient Characteristics

Hundred forty-four children with DCM were included in the study: 97 children (67%) diagnosed during the study period, and 47 patients (33%) diagnosed before the start of the study in 2010. Median age at diagnosis was 1.5 years [IQR 0.12–9.97], and 63 children (44%) were diagnosed before the age of 1 year.

Initial diagnostic categories included idiopathic DCM in 67 children (46%), myocarditis in 23 (16%), NMD in 7 (5%), familial DCM in 18 (13%), IEM in 4 (3%), malformation syndrome in 2 (1%), and “other” in 23 (16%). The “other” category included anthracycline-related DCM in 8 (6%), LV dilation and systolic dysfunction with non-compaction cardiomyopathy (NCCM) in 6 (4%) (as described by van Waning et al^18^), DCM based on tachyarrhythmia in 3 (2%), LV infarction in 2 (1%), vasculitis in 2 (1%), and congenital AV-block in 1 (1%).

The median follow-up time was 2.1 years [IQR 1.0–4.3]. Table 1 describes the clinical characteristics of the cohort.

**Table 1.**
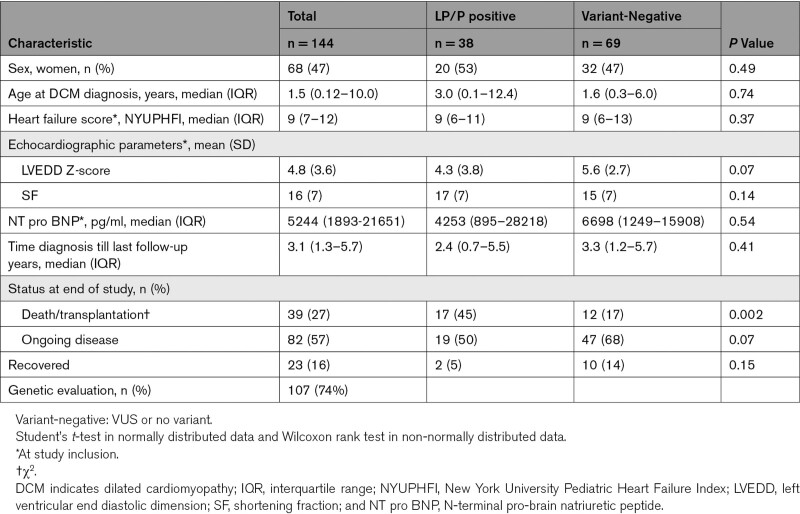
Characteristics of Children with Dilated Cardiomyopathy Stratified by LP/P Variant-Positive Patients And Variant-Negative Patients

### Genetic Findings

Hundred-seven of 144 DCM patients (74%) underwent genetic testing, with some patients undergoing >1 test. These 107 children were recruited from 105 families. In 1 family 2 siblings and a cousin were recruited. In another family, 2 sibs were recruited, but genetic testing was performed in only one. No difference was observed in the percentage of genetic testing in patients who reached the SE compared with those who did not (29 of 39 (74%) versus 78 of 105 (75%), *P* = 0.9). Sixteen (15%) patients underwent only Sanger sequencing of 1 or more genes, whereas 67 (63%) patients had a targeted NGS gene panel (including those who also had undergone Sanger sequencing and/or ES). Thirty-three patients (31%) had ES with analysis of an expanded gene panel related to cardiomyopathy, 1 patient had genome sequencing with comprehensive analysis of all known genes and DNA of 3 patients was analyzed with another technique (SNP-array, multiplex PCR and southern blot analysis, multiplex ligation-dependent probe amplification). For 3 patients, information on which technique had been used could not be retrieved. In at least 58 patients (54%), all cardiomyopathy-related core genes had been sequenced and analyzed.^19^ *MYH7* had been tested in 92 patients (86%) (see also Supplemental Tables I–III and Supplemental Figure 1). Table 2 describes the number of genetically evaluated patients and the number of likely pathogenic or pathogenic (LP/P) variants per diagnostic category.

**Table 2.**
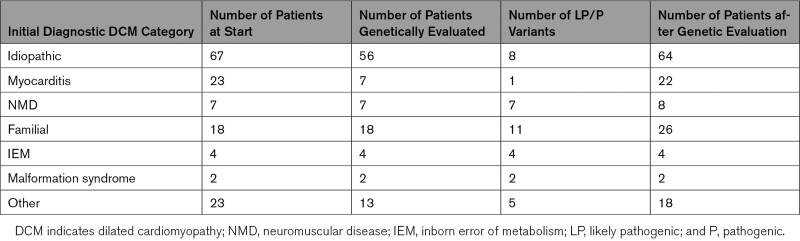
Genetic evaluation and Outcome Per Diagnostic Category

**Figure 1.**
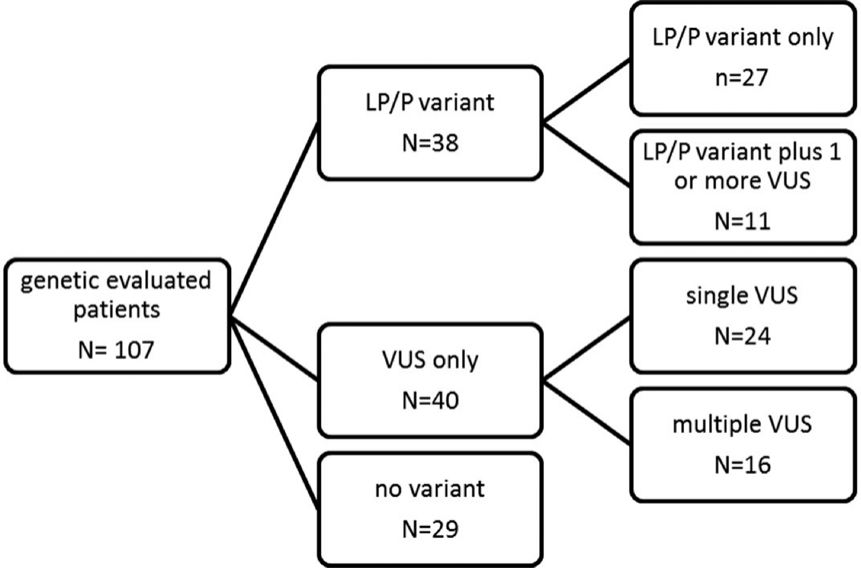
**Outcome genetic evaluation.** The cases that were not genetically tested are explained by the underlying diagnosis, which was not an indication for a genetic test improbable at the time of initial evaluation: 16 cases of myocarditis, 6 cases of chemotherapy-related dilated cardiomyopathy (DCM), 2 cases of left ventricular-infarction, and 1 tachyarrhythmia-induced DCM. In the remaining 12 cases (11 idiopathic DCM and 1 DCM with features of non-compaction), genetic testing was declined by the parents for varying reasons. LP/P variant indicates likely pathogenic or pathogenic (class 4 or 5 variant according to the American College of Medical Genetics (ACMG) classification); and VUS, variant of unknown significance (class 3 variant according to the ACMG classification).

Thirty-eight (36%) patients carried a LP/P variant, including 11 who had 1 or more additional VUSs. Forty patients (37%) had only 1 or more VUS, whereas 29 (27%) patients had no variant (Figure 1). The variant identified in 3 patients recruited from 1 family was classified as VUS.

LP/P variants were found in 21 different genes, with *MYH7* the largest contributor of pathogenic variants (8 LP/P variants (21%)). The second highest contributors were *TTN* and *TPM1*, each accounting for 8% of positive test results. No variants were identified in a number of cardiac genes that are part of standard gene panels (Supplemental Table I). The clinical and genetic characteristics of the 38 patients with an LP/P variant are described in Table 3. Two patients with LP/P variants in genes related to a malformation syndrome (Alström syndrome) were found. Seven patients had LP/P variants in genes related to NMD (Duchenne disease, Becker disease, infantile type I muscle fiber disease and cardiomyopathy, centronuclear myopathy type 5). Four had LP/P variants in genes related to IEM (Very Long Chain Acyl-CoA dehydrogenase Deficiency, propionic acidemia, Barth syndrome, and GM1 gangliosidosis). In all patients, clinical phenotype, disease course and/or muscle biopsy results, and/or additional urine and enzyme analyses were compatible with the genetic diagnosis. In at least 1 patient, the genetic diagnosis led to new unsuspected clinical findings; in the patient with Barth syndrome, neutropenia and skeletal myopathy were subsequently diagnosed. In 3 patients, we found LP/P variants in 2 genes: *MYH7*/*RYR2, SCN5A*/*KCNQ1*, and *TBX20/GLB1*. DCM-associated *SCN5A* variants have been shown to have either loss- or gain-of-function effects on cardiac sodium channel activity.^20–22^ In 1 patient, biallelic *ASNA1* variants were found (as described previously^23^). We also found 6 LP/P variants in 4 patients that we did not deem to be explanatory for the DCM, including 2 compound heterozygous truncating variants in *CEP135*, a *de novo* deletion of chromosome 14q22.3q23.1 (Hg19: 57,007,506-61,613,506), 2 compound heterozygous pathogenic missense variants in *SLC37A4*, and a *de novo* missense variant in *MAP3K7*. Six of 38 variants (15%) were proven *de novo* (in 1 patient with a pathogenic variant in *TNNT2*, no data on segregation were available).

**Table 3.**
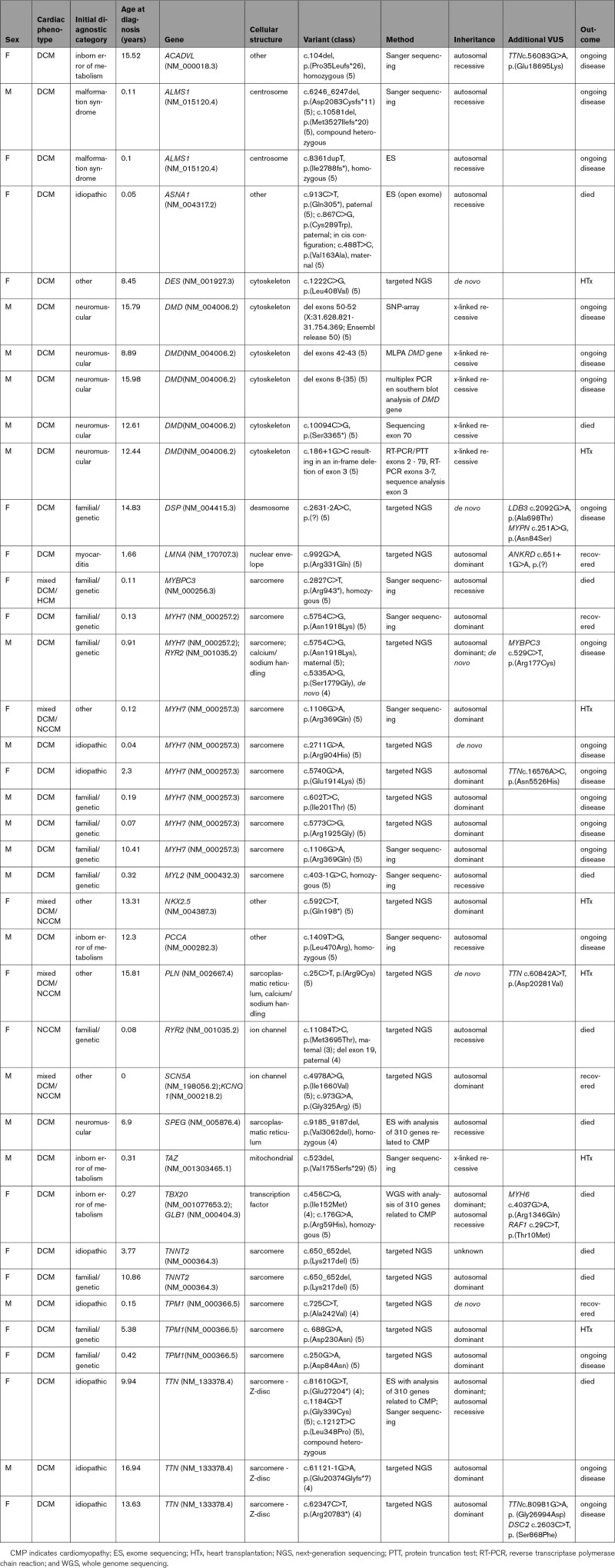
Clinical and Genetic Characteristics of Patients with Identified LP/P Variants

Cardiac screening of first-degree relatives was performed in 57 families (57/105; 54%). In 22 families (22/57; 39%), 1 or more relatives were affected (in 17 cardiomyopathy, in 3 rhythm disorder, and in 2 families SCD at age < 35 years). In 21 families, cardiac screening was not performed or not advised, and in 27 families it was unknown whether cardiac screening had taken place and/or results were not known. In 13 of the 38 families in which an LP/P variant was found (13/38; 34%), a cardiac phenotype was diagnosed in a relative (4 siblings in case of recessive disease, 9 first-degree relatives in case of dominant disease). However, it is not known whether this followed carrier screening or clinical phenotyping had been performed before genetic testing.

The diagnostic classifications of 20 patients changed during the study period. LP/P variants were found in 8 children (12%) who had initially been diagnosed with idiopathic DCM, and their cases were therefore reclassified to familial/genetic DCM. Seven of the 23 patients who were diagnosed with myocarditis underwent genetic evaluation, and a pathogenic *LMNA* variant was found in 1 patient. The diagnostic category of this patient was therefore reclassified as familial/genetic DCM (Table 2). In 5 patients with LV dilation and systolic dysfunction with NCCM classified as “other,” an LP/P variant was found (*DES, MYH7, NKX2.5, PLN, SCN5A* (Table 3)). One patient initially classified as familial was reclassified as NMD after genetic evaluation (*MYL2*). One patient with phenotypical muscular dystrophy, in whom the diagnosis was confirmed by muscle biopsy, had negative genetic findings. The classification of this patient was maintained as NMD.

In addition, variant reclassification altered the definitive diagnosis in 5 patients (26% of all diagnostic reclassifications, Table 3). In these patients with a putative LP/P variant leading to allocation into the familial/genetic DCM group, the variant was reclassified as a VUS and patients were reclassified as idiopathic DCM. None of the variants initially classified as VUS were reclassified as LP/P (Table 2).

### Clinical Outcome

During the study period, 39 patients (27%) reached SE: 17 patients died (12%) and 22 patients (15%) underwent HTx. Median time from diagnosis to death was 0.09 years [IQR 0.03–1.1]. Median time to HTx was 2.9 years [IQR 1.1–6.1]. At the end of the study, 23 children (16%) had recovered (35% diagnosed with myocarditis), but 82 children (57%) had ongoing disease.

### Association of LP/P Variants with Clinical Outcome

Seventeen of 38 children with a LP/P variant reached SE, whereas 19 had ongoing disease and 2 (with variants in *MYH7* and *LMNA*) recovered.

Children with a LP/P variant were more likely to die or undergo HTx compared with children without a pathogenic variant (17 of 38 (45%) versus 12 of 69 (17%), *P* = 0.002). We found no differences in clinical characteristics at time of diagnosis between children with a LP/P variant and those without (Table 1). Median age at SE tended to be lower in children with a LP/P variant, however this difference was not statistically significant (*P* = 0.19). Median age at SE was 10.9 years [IQR 0.6–16.1] in variant-positive patients, and 5 of 17 (29%) were under 1 year of age. In variant-negative patients, median age at SE was 13.3 years [IQR 6.9–14.6], and the age of the youngest patient at SE was 3.5 years.

Of the 17 LP/P variant-positive children reaching SE, 10 (53%) died and 7 (41%) underwent HTx. The majority of variant-negative children who reached a SE underwent HTx (8/12; 67%), while 4 of 12 children died (36%, *P* = 0.26).

**Figure 2.**
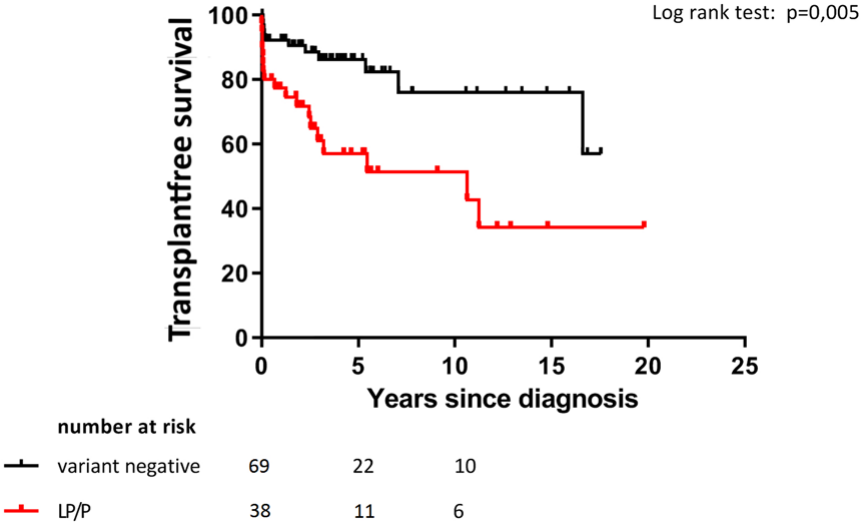
Kaplan-Meier analysis of 107 genetically evaluated children with dilated cardiomyopathy, children with a likely pathogenic or pathogenic (LP/P) variant versus no variant or variant of unknown significance LP/P: class 4 or 5 variant according to the American College of Medical Genetics classification Transplant-free survival (%) since diagnosis in LP/P group versus variant negative group.

Transplant-free survival was significantly lower in patients with a LP/P variant compared with variant-negative patients (*P* = 0.006, Figure 2). This was also true when excluding the 8 children who were clinically and/or genetically diagnosed with NMD (*P* = 0.04). Children with a LP/P variant had a 2.8-times increased risk of death or HTx (hazard ratio 2.8; 95% CI 1.3–5.8, *P* = 0.007). This hazard ratio stayed identical when excluding those diagnosed with NMD (2.8; 95% CI 1.2–6.0, *P* = 0.01). Transplant-free survival was higher in *MYH7*-positive children compared with those with a LP/P variant in other genes (*P* = 0.03, KM curve not shown).

In children without LP/P variants, we did not find an association between the presence or absence of VUSs and SE: 6/40 with one or more VUSs reached a SE versus 6/29 without VUSs reached a SE (*P = 0*.4, Figure 1).

The number of patients reaching a SE and the heterogeneity of genetic findings meant that we had insufficient statistical power to explore the relationship between single affected genes or *de novo* variants and outcome.

## Discussion

In this cohort of 107 genetically evaluated children with DCM, 38 children (36%) carried a LP/P variant in a DCM-related gene, most often in *MYH7.* Children with DCM who carried a LP/P variant had a 2.8-times increased risk of death or HTx compared with children without such a variant, but clinical characteristics at time of diagnosis did not differ between the 2 groups. In addition, children with a LP/P variant were more likely to die or undergo HTx at an earlier age. These findings highlight the importance of early genetic testing in children with DCM, as the determination of a genetic cause can be valuable for predicting clinical outcome.

### Yield of Genetic Testing in Children with DCM

In adults with DCM, the yield of genetic testing varies between 16% and 37%.^24^ There are only a few studies on current genetic testing in pediatric DCM. These studies differ in inclusion criteria (isolated DCM versus non-isolated DCM), the extent of genetic testing, and variant filtering and interpretation. Pugh et al reported an overall yield of 37% in 766 individuals with DCM (including 286 patients younger than 18 years) using gene panels that included between 5 and 46 genes, with *TTN* being the largest contributor (up to 14%). Selecting for all patients aged <18 years, a yield of 15.4% can be distracted.^10^ Kühnish et al found 8 LP/P variants in 34 pediatric patients with DCM (24%) using a panel‐based NGS approach targeting 89 genes, which identified variants in *TTN* (1), *TNNT2* (2), *TNNI3* (1), *MYH7* (1), *MYBPC3* (1), and *ACTC1* (1).^25^ In a recent study by Herkert et al, combining copy number variant analysis with stepwise trio-based ES yielded a diagnosis in >50% of pediatric DCM patients. They also identified LP/P variants in patients with nonfamilial DCM and in patients with extra-cardiac features or possible myocarditis.^7^ In a study by Long et al, ES in 18 families with DCM (including 3 syndromic cases) yielded a genetic diagnosis in 33% when filtering for 55 known DCM genes. When they expanded their analysis by filtering the exome for compound heterozygous and *de novo* variants, they diagnosed 4 additional patients, including carriers of rare and syndromic genes (*ALMS1* and *PRDM16*) and 2 potential novel genes (*RRAGC* and *TAF1A*), resulting in a final yield of 50%. Vasilescu et al reported a genetic diagnosis in 10 of 37 pediatric DCM patients (27%), of which 3 were in novel or less-established disease genes (*PPA2, TAB2*, and *NRAP*) and 2 were in mitochondrial DNA. They also showed an increase in a genetic diagnosis with later onset of cardiomyopathy: age <1 year – 34% positive DNA diagnosis, age 1 to 5 years – 38% and age >6 years – 60%. They further showed that infants manifesting before 1 year of age had the poorest prognosis, especially when their cardiomyopathy was associated with a metabolic or syndromic origin, which is in line with previous studies.^9^ Finally, in a study in neonates with heart failure, ES was diagnostic in 10 of 15 (68%), although only 20% had a clinical diagnosis of DCM.^26^

The yield of genetic testing in pediatric DCM is consistent with that in adult-onset non-ischemic DCM, varying between 19% and 54%.^7,8,10,27–29^ However, the spectrum of genes involved in pediatric DCM differs from that seen in adult DCM.^30,31^ Pugh et al showed that the genes implicated in DCM vary with age. In adults and children 2–18 years of age, the majority of variants were located in *TTN* and *DSP*. In children under 2 years of age, *MYH7* was predominantly mutated and no *TTN* variants were found.^10^ This matches our findings as 6 of 8 children in our cohort with LP/P *MYH7* variants were under the age of 1 year at diagnosis, confirming that these variants frequently underlie DCM with infant presentation.

Our yield of 36% LP/P variants in pediatric DCM and the spectrum of genes involved are thus in line with international literature, but also leave room for further increase in yield, e.g. by systematically offering ES with analysis of an expanded gene panel or of all disease-associated genes. This is especially important for children <1 year of age at diagnosis, where the diagnostic yield of ES goes up to 55% (5/9) and 3 of 5 (60%) genes with pathogenic variants were not part of (adult-onset) cardiomyopathy panels because they are involved in metabolic and syndromic diagnoses.^26^

### The Predictive Value of the Variants Detected

In adults with non-ischemic DCM, there is increasing insight into the association between certain pathogenic variants and outcome. In a large meta-analysis, the highest HTx rate was found in *LMNA* mutation carriers (27%), while *RBM20* mutation carriers underwent HTx at a younger age (mean 28.5 years) than carriers of pathogenic variants in other genes (mean 41–43 years).^32^ Another study in 5267 individuals, ranging from healthy volunteers to end-stage DCM patients, showed that *TTN*-truncating variant-positive DCM patients reached the SE of death, HTx or ventricular assist device at earlier ages and sooner after enrolment than *TTN*-truncating variant-negative DCM patients.^33^ Janswijer et al found that truncating *TTN* mutations were associated with a milder form of DCM compared with that seen in patients with *LMNA* variants or idiopathic DCM.^34^ These findings were (partly) explained by differences in disease severity and the number of adverse events between the cohorts. In a study on the prognosis of 52 adults with DCM carrying rare variants in sarcomeric genes (*MYH6, MYH7, MYBPC3, TNNT2*, and *TTN*), it was shown that death/HTx‐free survival dramatically decreased after 50 years of age in variant-positive patients compared with variant-negative patients.^35^

When predicting outcome in children with DCM, the genetic contribution is less clear. Specific variants (eg, in *LMNA* or *SCN5A*) have been linked to sudden cardiac death, as this is related to malignant ventricular arrhythmias.^8,36^ However, sudden cardiac death in children with DCM (5-year incidence of 3%) is a less prevalent clinical issue than death due to heart failure or the need for HTx.^37^ To the best of our knowledge, only 1 study has systematically evaluated children with non-HCM cardiomyopathy (with 56 of 70 patients diagnosed with DCM) for genetic disease and association with outcome: HTx were more often performed in variant-positive children than in variant-negative subgroups (48% versus 34%), and the variant-positive children had higher mortality (17% versus 2%). Of note, outcome was not specified for the 56 DCM patients.^38^

In our study, which reports on the largest cohort of genetically tested children with DCM to date, we also observed decreased survival in children with a LP/P genetic variant. Our study is thus an important contribution to the mounting evidence that carrying a LP/P variant puts children at an increased risk of death or HTx. We also found that *MYH7* variant carriers were less likely to die or undergo HTx compared with patients with LP/P variants in other genes, although our numbers were small. Whether this truly implies that *MYH7* variants are relatively benign remains unknown. In adult DCM studies such a favorable genotype–phenotype relation could not be demonstrated for *MYH7* compared with *LMNA, PLN, RBM20, MYBPC3, TNNT*2, and *TNNI3* (29).

### Clinical Implications of Our Study

The results of our study justify incorporating genetic testing early on in the diagnostic work-up of all children with DCM.^7^ Since determining disease pathogenesis is essential for prognosis and counseling, genetic testing should be offered as soon as possible after diagnosis. In both adults and children, there is also increasing evidence that the presence of external causal factors (eg, chemotherapy or myocarditis) does not preclude a genetic cause for the DCM.^39–41^ The diagnosis of myocarditis is often only based on clinical characteristics.^42^ In our study we identified a pathogenic *LMNA* variant in a child diagnosed with myocarditis, which is a good example of how previously silent genetic defects might predispose to heart failure early in life when viral myocarditis acts as a second hit.^43^ A number of experts have pointed out the potential relevance of underlying genetic abnormalities, that may be unmasked or present in the setting of a clinical picture resembling myocarditis or even in patients with biopsy-proved myocarditis.^44^ This is further supported by a recent study from Seidel et al, in which they found likely pathogenic or pathogenic variant in 22% of patients with biopsy-proved myocarditis, especially in the group of patients with myocarditis mimicking DCM.^45^ These findings suggest to genetically evaluate all children diagnosed with DCM in an early stage, regardless of presumed disease pathogenesis. They also call for genetic re-evaluation of all children with DCM who have been tested previously, but to whom ES has not been offered. In our cohort, 74% percent of children were genetically evaluated, which clearly leaves room for improvement. Optimizing collaboration between pediatric cardiologists and clinical geneticists and genetic counseling of parents might increase the uptake of genetic testing.

At present, the direct translation of a genetic variant into individual clinical risk prediction in the pediatric population is challenging. DCM in children is characterized by genetic heterogeneity, a situation contrary to that for HCM, where a relatively limited number of genes seem to be involved, predominantly those related to the sarcomere.^46^ Penetrance and age of presentation also vary in DCM.^47–49^ Based on our findings, children who carry an LP/P variant in an established gene associated with DCM should be considered at an increased risk for adverse outcomes. The use of genetic information for better management and risk prediction will require close collaboration between research centers and analysis of pooled data. In this respect, the results of the “PCM Genes study” of the PCMR, which aims to offer ES to 600 children with DCM, will provide more insight into genetic testing and associations with outcome.^50^ As long as it is not clear how to differentiate between recovery and remission, children who are variant-positive but recover should continue to receive follow-up care.^51^

## Limitations

Our study has limitations. Initially, diagnostic categories were assigned following the etiologic categories of the PCMR.^11^ However, these data pre-dated many clinically available genetic tests and, along with other epidemiologic studies, highlight the uncertain etiological basis of cardiomyopathy given that the majority of DCM patients were identified as idiopathic. Future directions of the registry include the use of ES to improve diagnostic strategies, which may lead to different etiologic classification.^50^ In our study, several idiopathic cases turned out to be familial/genetic. Furthermore, IEM and NMD are considered distinct categories even though a genetic diagnosis typically underlies these diseases as well. For that reason, we have included IEM and NMD as LP/P variant-positive in our analyses. It might have been more transparent if we had assigned 1 genetic diagnostic category that included subcategories of IEM and NMD in addition to those with a LP/P variant in an explanatory gene and those with 2 or more affected first-degree family members. Secondly, some of our data were retrospective, and missing data might be an issue here despite the efforts we made to obtain all available data. Thirdly, genetic testing panels changed during the study period, which influences genetic yield. Furthermore, we could only be certain that a variant was *de novo* in cases with genetic evaluation of both parents, so the true number of *de novo* cases and possible relation to worse outcome remains unknown. Finally, our sample size was too small to test associations between individual variants/genes and phenotype.

## Conclusions

Genetic testing is a valuable tool predicting outcome in children with DCM and counseling families. Patients with a LP/P variant have an overall worse prognosis. Genetic testing should therefore be incorporated in clinical care of all children with DCM, regardless of presumed disease pathogenesis.

## Article Information

### Acknowledgments

The authors thank the patients and their families for their participation, research nurses Badies Manaï and Annelies Hennink for data collection, and Kate McIntyre for editing the article.

### Sources of Funding

MH van der Meulen was supported by a joint grant from “Stichting Hartedroom” [Rotterdam, the Netherlands] and the “Netherlands Heart Foundation” (2013T087). JP van Tintelen and M Dalinghaus acknowledge the support from the Netherlands Cardiovascular Research Initiative, an initiative with support of the Dutch Heart Foundation (CVON2014-40 DOSIS; CVON2020B005 DOUBLE-DOSE).

### Disclosures

None.

### Supplemental Material

Supplemental Methods

Supplemental Table S1 (Data sets)

Supplemental Tables S2-S3

Supplemental Figure S1

References^52–57^

## Supplementary Material


