# Supplementary material for: Genetic Evaluation of A Nation-Wide Dutch Pediatric DCM Cohort: The Use of Genetic Testing in Risk Stratification
Source: Circ Genom Precis Med. 2022 Sep 30;15(5):e002981. doi: 10.1161/CIRCGEN.120.002981 (PMC9622377; doi:10.1161/CIRCGEN.120.002981)

SUPPLEMENTAL MATERIAL

Supplemental Methods

Patients

Data were collected in a multicenter, observational study design. Eight tertiary pediatric cardiology centers cooperated in the study, as previously described [52]. These eight centers serve almost 100% of the Dutch pediatric population.

Patients (age < 18 years at diagnosis) were included from October 2010 to July 2017. In addition, we retrospectively enrolled children diagnosed with DCM before 2010. The first member of each family who presented to our services with a diagnosis of DCM was designated the proband for this analysis.

DCM was defined as the presence of impaired systolic function (fractional shortening (FS) ≤25%) and left ventricular (LV) dilation (LV end-diastolic dimension (LVEDD) *z*-score >2 for body surface area) [53,54]. Patients with additional structural heart disease that explained their LV dilation were excluded. A diagnostic work-up was performed in all patients, as previously described [11,55]. Patients were subsequently classified into an initial diagnostic category within the six months following their DCM diagnosis. Diagnostic categories consisted of: idiopathic, myocarditis, neuromuscular disease (NMD), familial, IEM, malformation syndrome or other. This classification follows the standard of the Pediatric Cardiomyopathy Registry (PCMR) [11,50]. Familial DCM is defined as two or more affected family members and/or an explanatory genetic finding.

Diagnosis of myocarditis was made based on clinical grounds and viral test results. Myocarditis was 'definite' if there was histological or immune-histological evidence of myocarditis. Myocarditis was 'probable' when blood plasma/serum or cerebrospinal fluid PCR or culture was positive for enterovirus, adenovirus, parechovirus or human parainfluenza virus, or if blood plasma/serum PCR or culture was positive for parvovirus B19, HHV6, cytomegalovirus or Epstein-Barr virus accompanied by serological proof of a primary infection (seroconversion and/or positive IgM) [55]. In the patients

diagnosed before 2010, data on diagnostic work-up, family history and genetic variant segregation analysis were retrospectively collected.

Study endpoint (SE) was defined as all-cause death or heart transplantation (HTx). In addition, patient status at the last follow-up visit was recorded as ‘ongoing disease’ or ‘recovered’. Recovered was defined as two consecutive echocardiograms with normalized LVEDD and FS, with the date of the first normalized echocardiogram considered the date of recovery. Furthermore, gender, age at diagnosis, New York University Pediatric Heart Failure Score (NYUPHFI), NT-proBNP, and standardized echocardiogram (LVEDD, FS) were recorded at inclusion. All study data were collected during routine outpatient clinic visits or hospital admissions. Subjects were followed until SE was reached, the age of 18 years, or the last outpatient visit within the study window. This study was approved by the Medical Research Ethical Committee of the Erasmus University Medical Center (MEC 2014-062) and performed in accordance with the Declaration of Helsinki.

Genetic evaluation and variant classification

The genetic data we collected were obtained retrospectively and reflect the genetic evaluation that was common practice at that time: single gene testing (e.g., Sanger sequencing of *MYH7*), targeted next-generation sequencing (NGS) of a gene panel (range 28-70 genes), exome sequencing (ES) with analysis of genes related to cardiomyopathy [7] or open exome analysis. Additional genetic testing (e.g., SNP-array) was performed in a subset of patients in whom a malformation syndrome was suspected. As this was an observational study, patients who had no genetic testing or a test that is now considered too limited were not actively referred for genetic (re)evaluation.

Patients were considered genetically evaluated when at least one genetic test was performed. The pathogenicity of the variants was assessed using Alamut Visual Software (Interactive Biosoftware, Rouen, France). All variants were reclassified (December 2019) by a molecular geneticist specialized in cardiogenetics (RLdD) according to ACMG criteria [56]. Variants with a minor allele frequency <0.1% in the Genome Aggregation Database (gnomAD) were considered rare. Nonsense and frameshift

variants were considered null variants if they occurred proximal to the last 50 bases of the penultimate exon. We defined two groups: patients with a likely pathogenic (LP) or pathogenic (P) variant (class 4 or 5) and patients without a pathogenic variant, including patients with one or more variants of unknown significance (VUS, class 3) [57].

Statistical analysis

Categorical variables were reported as numbers and percentages. Continuous variables were reported as means with standard deviation (SD) when normally distributed, or as medians with interquartile range (IQR) when non-normally distributed. To compare clinical characteristics between patients with a LP/P variant and those with negative genetic test results, the student's t-test was used in case of normally distributed variables and the Wilcoxon rank test was used when variables were non-normally distributed. A Chi-square test or two-sided Fisher exact test was performed to examine the relation between categorical data.

We used the Kaplan-Meier method to estimate transplant-free survival in the two groups. The log-rank test was used to determine whether the difference between the two curves was statistically significant. Univariate Cox regression analysis was used to test the predictive value of a LP/P variant. Proportional hazard assumptions were tested, and were not violated. The hazard ratio and 95% confidence interval (CI) were calculated. Testing was performed two-sided, and statistical significance was set at $P < 0.05$. All analyses were performed using IBM SPSS Statistics for Windows, version 24 (IMB Corp, Armonk, NY, USA).

Supplemental Tables

Supplemental Table II. Variants of unknown signficance identified in cardiomyopathy-associated genes.

Gene	Transcript	Patient	Nucleotide change	Protein change	Classification
ALPK3	NM_020778.4	P130	c.5155G>C	p.(Ala1719Pro)	VUS
ANKRD	NM_014391.2	P139	c.642_643delinsT	p.(Arg215Glufs*13)	VUS
	NM_014391.2	P98	c.651+1G>A	p.(?)	VUS
CAV3	NM_033337.3	P66	c.233C>T	p.(Thr78Met)	VUS
DMD	NM_004006.2	P63	c.1536C>A	p.(His512Gln)	VUS
	NM_004006.2	P96	c.9955T>C	p.(Cys3319Arg)	VUS
DSC2	NM_024422.4	P47	c.2603C>T	p.(Ser868Phe)	VUS
DSP	NM_004415.3	P136	c.2906C>T	p.(Thr969Ile)	VUS
DTNA	NM_001390.4	P117	c.*5A>G		VUS
	NM_001390.4	P81	c.153C>G	p.(His51Gln)	VUS
	NM_001390.4	P81	c.239G>A	p.(Arg80His)	VUS
	NM_001390.4	P20	c.784C>T	p.(His262Tyr)	VUS
FLNC	NM_001458.4	P143	c.4413A>T	p.(Gln1471His)	VUS
JUP	NM_002230.2	P33	c.778G>A	p.(Ala260Thr)	VUS
	NM_002230.2	P103	c.1571T>C	p.(Ile524Thr)	VUS
LAMA4	NM_001105206.2	P126	c.4345C>T	p.(Arg1449Trp)	VUS
	NM_001105206.2	P120	c.5361_5365del	p.(Lys1787Asnfs*11)	VUS
LDB3	NM_007078.2	P81	c.1051A>G	p.(Thr351Ala)	VUS
	NM_007078.2	P6	c.2092G>A	p.(Ala698Thr)	VUS
	NM_001080114.1	P37	c.433G>A	p.(Gly145Ser)	VUS
MYBPC3	NM_000256.3	P129	c.529C>T	p.(Arg177Cys)	VUS
	NM_000256.3	P149	c.852-20C>A	p.(?)	VUS
MYH6	NM_002471.3	P140	c.4037G>A	p.(Arg1346Gln)	VUS
	NM_002471.3	P81	c.4193G>A	p.(Arg1398Gln)	VUS
MYH7	NM_000257.3	P100	c.2981A>G	p.Lys994Arg	VUS
	NM_000257.3	P92	c.495G>A	p.(Met165Ile)	VUS
	NM_000257.3	P18	c.495G>A	p.(Met165Ile)	VUS
	NM_000257.2	P52	c.784G>A	p.(Asp262Asn)	VUS
	NM_000257.2	P31	c.2890G>C	p.(Val964Leu)	VUS
MYOZ2	NM_016599.4	P18	c.148C>T	p.(Leu50Phe)	VUS
MYPN	NM_032578.3	P97	c.59A>G	p.(Tyr20Cys)	VUS
	NM_032578.2	P6	c.251A>G	p.(Asn84Ser)	VUS
NEXN	NM_144573.3	P82	c.1174C>T	p.(Arg392*)	VUS
PKP2	NM_004572.3	P135	c.184C>A	p.(Gln62Lys)	VUS
	NM_004572.3	P20	c.1415C>T	p.(Pro472Leu)	VUS
	NM_004572.3	P114	c.2083C>T	p.(Arg695Cys)	VUS
	NM_004572.3	P135	c.2258T>C	p.(Leu753Pro)	VUS
PRDM16	NM_022114.3	P79	c.1882G>A	p.(Asp628Asn)	VUS
RAF1	NM_002880.3	P140	c.29C>T	p.(Thr10Met)	VUS
RBM20	NM_001134363.2	P12	c.2381G>A	p.(Arg794Lys)	VUS
	NM_001134363.2	P37	c.2381G>A	p.(Arg794Lys)	VUS
	NM_001134363.2	P52	c.2761A>T	p.(Ile921Phe)	VUS

SCN5A	NM_198056.2	P81	c.2512G>A	p.(Ala838Thr)	VUS
TLL1	NM_012464.4	P31	c.2087A>C	p.(Glu696Ala)	VUS
TNNC1	NM_003280.2	P146	c.47A>G	p.(Gln16Arg)	VUS
	NM_003280.2	P114	c.202G>A	p.(Gly68Ser)	VUS
	NM_003280.2	P101	c.205A>G	p.(Ser69Gly)	VUS
	NM_003280.2	P81	c.304C>T	p.(Arg102Cys)	VUS
TTN	NM_133378.4	P1	c.8707T>C	p.(Cys2903Arg)	VUS
	NM_133378.4	P24	c.76645G>A	p.(Val25549Ile)	VUS
	NM_001267550.2	P103	c.13048G>A	p.(Val4350Met)	VUS
	NM_001267550.2	P48	c.16576A>C	p.(Asn5526His)	VUS
	NM_001267550.2	P91	c.34283C>T	p.(Pro11428Leu)	VUS
	NM_001267550.2	P33	c.35189_35191del	p.(Glu11730del)	VUS
	NM_001267550.2	P138	c.46054G>A	p.(Asp15352Asn)	VUS
	NM_001267550.2	P116	c.56083G>A	p.(Glu18695Lys)	VUS
	NM_001267550.2	P113	c.59189C>T	p.(Ala19730Val)	VUS
	NM_001267550.2	P104	c.66820C>T	p.(Arg22274Cys)	VUS
	NM_001267550.2	P113	c.73994C>T	p.(Thr24665Met)	VUS
	NM_003319.4	P64	c.20047A>C	p.(Asn6683His)	VUS
	NM_003319.4	P64	c.43331T>G	p.(Val14444Gly)	VUS
	NM_003319.4	P143	c.8604G>T	p.(Trp2868Cys)	VUS
	NM_001267550.1	P95	c.43700G>C	p.(Arg14567Thr)	VUS
	NM_001267550.1	P95	c.55355C>G	p.(Ser18452Cys)	VUS
	NM_133378.4	P88	c.15125T>C	p.(	VUS
	NM_133378.4	P69	c.60842A>T	p.(Asp20281Val)	VUS
	NM_133378.4	P88	c.80887C>T	p.(Leu26963Phe)	VUS
	NM_133378.4	P47	c.80981G>A	p.(Gly26994Asp)	VUS
	NM_133378.4	P88	c.81029G>A	p.(Arg27010His)	VUS
	NM_133378.4	P88	c.89826T>A	p.(Asp29942Glu)	VUS

Supplemental Table III. Number of likely pathogenic or pathogenic variants normalized to frequency of analysis

Gene	Total	Number of LP/P variants	Percentage
MYH7	92	9	9,8
MYBPC3	90	1	1,1
LMNA	89	1	1,1
TNNT2	89	2	2,2
TPM1	88	3	3,4
TCAP	87		0,0

TNNI3	87		0,0
CSRP3	85		0,0
DES	85	1	1,2
EMD	85		0,0
LAMP2	85		0,0
LDB3	85		0,0
TAZ	85	1	1,2
ACTC1	84		0,0
GLA	84		0,0
MYL2	84	1	1,2
PLN	84	1	1,2
TTN	84	3	3,6
MYL3	83		0,0
TNNC1	83		0,0
VCL	83		0,0
BAG3	82		0,0
CAV3	82		0,0
CRYAB	82		0,0
PRKAG2	82		0,0
JPH2	80		0,0
MYPN	80		0,0
RBM20	80		0,0
SCN5A	80	1	1,3
ACTN2	79		0,0
ANKRD1	79		0,0
LAMA4	79		0,0
NEXN	79		0,0
CALR3	78		0,0
FHL1	78		0,0
MYOZ2	77		0,0
TTR	76		0,0
DSC2	75		0,0
DSG2	75		0,0
DSP	75	1	1,3
JUP	75		0,0
PKP2	75		0,0
ABCC9	75		0,0
MYH6	73		0,0
CTNNA3	70		0,0
MIB1	70		0,0
TMEM43	68		0,0
SGCD	66		0,0

FKTN	62		0,0
RYR2	61	2	3,3
DMD	60	5	8,3
DTNA	59		0,0
ILK	59		0,0
MYOZ1	58		0,0
PRDM16	57		0,0
MYLK2	55		0,0
CASQ2	53		0,0
FLNC	53		0,0
EYA4	51		0,0
GATAD1	51		0,0
PDLIM3	51		0,0
TBX20	51	1	2,0
TGFB3	48		0,0
NEBL	46		0,0
TXNRD2	45		0,0
ANO5	44		0,0
HCN4	44		0,0
TMPO	44		0,0
ACAD9	36		0,0
ALPK3	36		0,0
NKX2-5	36	1	2,8
RAF1	35		0,0
TRIM63	34		0,0
ACADVL	31		0,0
FKRP	31		0,0
ACTA1	30		0,0
AGL	30		0,0
GAA	30		0,0
PLEC	30		0,0
PNPLA2	30		0,0
SOD2	30		0,0
AARS2	29		0,0
AGK	29		0,0
ALMS1	29	2	6,9
CPT2	29		0,0
DNAJC19	29		0,0
KCNQ1	29	1	3,4
NDUFA1	29		0,0
NDUFA11	29		0,0
NDUFAF2	29		0,0

NDUFS1	29		0,0
NDUFS2	29		0,0
NDUFS3	29		0,0
NDUFS4	29		0,0
NDUFV1	29		0,0
NUBPL	29		0,0
PCCA	29	1	3,4
PSEN1	29		0,0
PSEN2	29		0,0
SCO2	29		0,0
SDHA	29		0,0
SDHAF1	29		0,0
SGCA	29		0,0
SYNE1	29		0,0
TMEM70	29		0,0
ARSB	28		0,0
ATPAF2	28		0,0
BRAF	28		0,0
COA5	28		0,0
COX10	28		0,0
COX14	28		0,0
DOLK	28		0,0
EPG5	28		0,0
FAH	28		0,0
FHL2	28		0,0
GLB1	28	1	3,6
HADHA	28		0,0
HRAS	28		0,0
IDUA	28		0,0
KRAS	28		0,0
MAP2K1	28		0,0
MAP2K2	28		0,0
MUT	28		0,0
NDUFAF1	28		0,0
NDUFAF3	28		0,0
NDUFAF4	28		0,0
NDUFAF5	28		0,0
NDUFB3	28		0,0
NDUFS6	28		0,0
NDUFV2	28		0,0
NF1	28		0,0
NRAS	28		0,0

PCCB	28		0,0
PTPN11	28		0,0
RIT1	28		0,0
SHOC2	28		0,0
SLC22A5	28		0,0
SLC25A20	28		0,0
SOS1	28		0,0
SPEG	28	1	3,6
TNNI3K	28		0,0
TSFM	28		0,0
COX20	27		0,0
COX6B1	27		0,0
FXN	27		0,0
HADHB	27		0,0
HFE	27		0,0
IDH2	27		0,0
MLYCD	27		0,0
SLC25A4	27		0,0
CHRM2	26		0,0
FOXD4	26		0,0
HSPB7	26		0,0
MRPL44	26		0,0
MURC	26		0,0
SGCG	20		0,0
N2A	19		0,0
Novex-1	19		0,0
Novex-2	19		0,0
Novex-3	19		0,0
LAMA2	18		0,0
NEB	17		0,0
SGCB	17		0,0
APOA1	15		0,0
FLNA	15		0,0
ASNA1	14	1	7,1
CTF1	14		0,0
FLT1	14		0,0
HOPX	14		0,0
NFKB1	14		0,0
SYNM	14		0,0
TRIM54	14		0,0
TRIM55	14		0,0

Supplemental Figures

Supplemental Figure I. Genes versus the number of times it was tested in patients

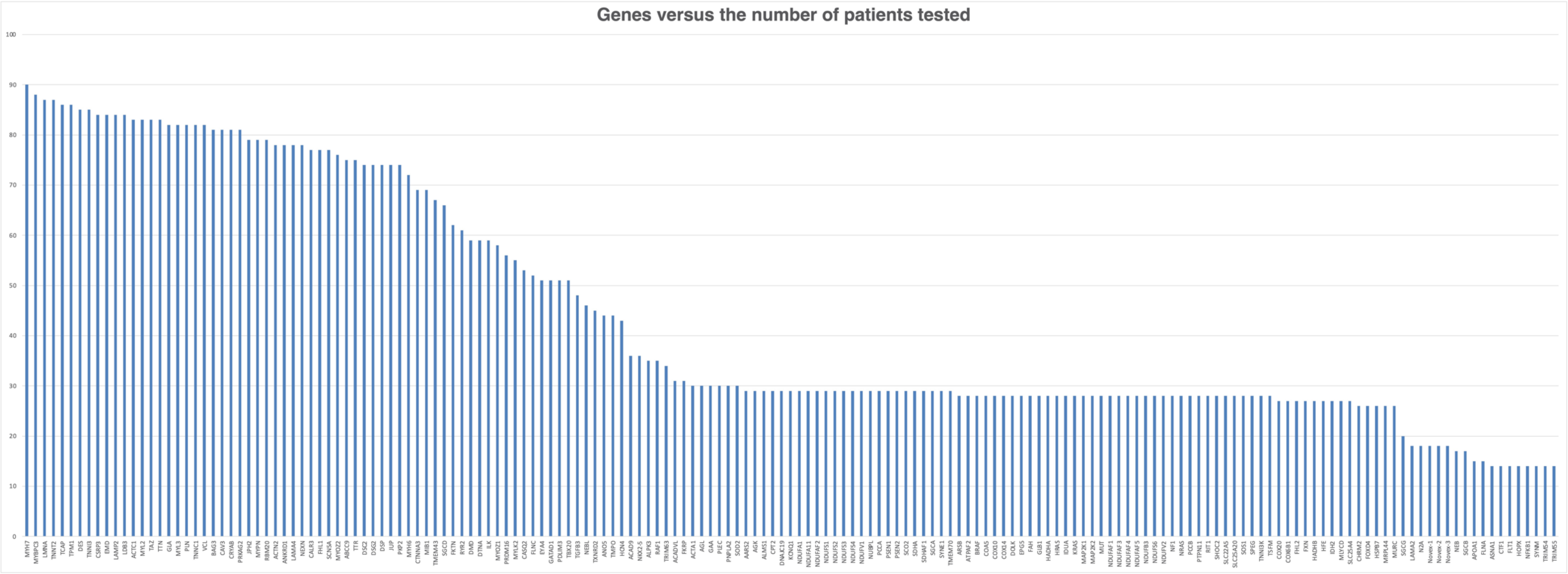

Supplement: Supplementary file 1 [file hcg-15-e002981-s001.pdf]
